# Technology Affordance in an Information and Communication Technology Delivered Group Psychotherapy and Exercise Program for Older People With Depressive Symptoms: A Multiple Triangulation Qualitative Study

**DOI:** 10.1093/geroni/igad063

**Published:** 2023-07-07

**Authors:** Dara Kiu Yi Leung, Frankie Ho Chun Wong, Edwin Lok Yan Wong, Lesley Sze, Melissa Chan, Tianyin Liu, Annabelle Pui Chi Fong, Wai Wai Kwok, Angie Kwan Yu Shum, Gloria Hoi Yan Wong, Terry Yat Sang Lum

**Affiliations:** Department of Social Work and Social Administration, The University of Hong Kong, Hong Kong, China; Department of Social Work and Social Administration, The University of Hong Kong, Hong Kong, China; Philip Merrill College of Journalism, University of Maryland, College Park, College Park, Maryland, USA; Department of Social Work and Social Administration, The University of Hong Kong, Hong Kong, China; Department of Social Work and Social Administration, The University of Hong Kong, Hong Kong, China; Department of Social Work and Social Administration, The University of Hong Kong, Hong Kong, China; Department of Social Work and Social Administration, The University of Hong Kong, Hong Kong, China; Department of Social Work and Social Administration, The University of Hong Kong, Hong Kong, China; Department of Social Work and Social Administration, The University of Hong Kong, Hong Kong, China; Department of Social Work and Social Administration, The University of Hong Kong, Hong Kong, China; Department of Social Work and Social Administration, The University of Hong Kong, Hong Kong, China; Department of Social Work and Social Administration, The University of Hong Kong, Hong Kong, China; Sau Po Centre on Ageing, The University of Hong Kong, Hong Kong, China

**Keywords:** Chronic pain, Cross-platform, Telehealth, Telemental health, Teletherapy

## Abstract

**Background and Objectives:**

Health and mental health interventions, such as psychotherapy and exercise programs, delivered via information and communication technology (ICT) may improve service access. However, adjustment among older people and in synchronous group interventions is more challenging. Technology affordance concerns the possibilities engendered by technology for various users and purposes and can help understand challenges in ICT-delivered groups and identify possible solutions.

**Research Design and Methods:**

Adopting a multiple triangulation approach, we observed ICT-delivered groups of acceptance and commitment therapy and exercise for older people with depressive symptoms, conducted focus groups with older people who had received group psychotherapy with or without an exercise component, and obtained clinical notes from interventionists. We conducted a thematic analysis of the observation notes, focus group transcriptions, and clinical notes.

**Results:**

Four focus groups were conducted with 22 participants (mean age = 72.6 years, standard deviation = 7.2, 86% female). We identified 3 challenges: (1) seeing–be seen dilemma, (2) speaking–hearing dilemma, and (3) blurred therapy–home boundary, and 2 solutions: (1) maneuvering layouts and collaborative tools, and (2) cross-platform mediated strategies. Participants struggled to observe the interventionist while simultaneously demonstrating their posture in front of a camera. Remaining silent and moderated turn-taking allowed for clearer hearing but limited interactions. Interruptions from the background environment and intersections of family living spaces disrupted audio-visual communication and jeopardized the sense of security. As a solution, interventionists maneuvered layouts and collaborative tools on teleconferencing applications to achieve intervention goals and provided support through different media.

**Discussion and Implications:**

The identified challenges and potential solutions can be understood from interactivity, portability, temporality, persistence, and multimediality. Technology affordance can guide ICT-delivered group design by matching the affordance of various technologies and communication media with the characteristics of the intervention and users to enhance efficacy and avoid an unnecessary digital divide.

**Translational Significance:** Delivering health and mental health interventions via information and communication technology is challenging among older people and in synchronous group format. By focusing on the functional and relational elements of the user-technology system, we identified unique and common challenges across intervention modalities, such as seeing–be seen dilemma and speaking–hearing dilemma, and potential solutions, such as cross-platform strategies. Interventionists can match the affordances of various information and communication technologies with intervention and participants’ characteristics to improve intervention efficacy and group cohesion while avoiding an unnecessary digital divide.

## Background and Objectives

Information and communication technology (ICT) has been employed in delivering health and mental health interventions, such as psychotherapy and exercise programs, to mitigate barriers and improve access to services (1). During the COVID-19 pandemic, preventive measures such as lockdown and social isolation not only led to loneliness, depression, anxiety, insomnia, and reduced level of physical activity among older people (2,3) but also limited their access to health and social services aimed at addressing these issues (4). ICT enables remote social connection and can serve as an alternative vehicle for mental health intervention when the therapists and participants are physically separated. Following the pandemic, interventionists have shifted from providing face-to-face interventions to synchronous online interventions. However, older people’s adjustment to synchronous group therapy appears more challenging and may require new knowledge and training, such as establishing therapeutic alliance and increasing group cohesion online (5). Understanding how ICT affords and constrains group therapeutic interactions can help optimize clinicians’ use of technology to improve intervention performance.

### Technology Affordance

With roots in perceptual psychology, Gibson theorized “affordance” as the potential options of action or purpose made available to or easier for a subject by another item. This initial conceptualization emphasized that affordances are both functional (ie, empowering or restricting activities) and relational (ie, different enabling effects for varying subjects) (6). Objects, whether natural or manmade, can derive affordance from both their inherent qualities and perceived applications (7). Hence, the affordance of a particular thing should be considered with the relevant individuals and wider social or technological contexts in mind, such as communal expectations and mechanical requirements. Nowadays, the popularized notion of “technology affordances” specifically refers to “the functional and relational elements of the user-technology system” (8). In other words, technology affordance concerns the possibilities engendered in practice by technologies for their various users and purposes.

### Technology Affordance in ICT-Delivered Groups

Research on ICT-delivered groups is limited, particularly those that aimed at older people who are likely affected by the digital divide (9). ICT-delivered groups comprise 2 major categories: synchronous groups, where all participants are online simultaneously, and asynchronous groups, where participants can connect to the group at different times (5). Synchronous groups are usually delivered as video conferences through platforms that allow for both audio and video communication (eg, Zoom, Skype). Conversely, asynchronous groups usually utilize Internet forums (eg, Google Forum) and instant message platforms (eg, WhatsApp) and are mainly based on text messages. Synchronous groups are of greater interest and have received growing adoption in telemental health services (5).

Recently, Yeshua-Katz and colleagues identified 4 technology affordances relevant to digital interventions: temporality, interactivity, multimediality, and portability, and recommended combining and matching affordances of different media to address intervention goals (10). These affordances were interpreted in terms of relative degree. For example, videoconferencing’s fixed and time-limited nature (ie, low temporality) facilitates the delivery of a planned semi-structured intervention protocol, whereas messaging application allows flexible and continuing communication outside the intervention session (ie, high temporality) that is crucial to a successful intervention. The high interactivity in videoconferencing and messaging applications has also been suggested to encourage connectedness and socialization.

Older people participating in a videoconferencing-delivered exercise group felt a sense of belonging and familiarity with the group (11). They also exhibited higher adherence to the program than those who received remote support for independent physical activity (12). However, there is also evidence suggesting that therapeutic alliance or group cohesion may not be adequately represented in psychotherapy and medical support groups delivered via video teleconference (13). Participants felt it more challenging to connect with other members in the virtual environment because of a lack of group participation and reported lower group cohesion than those in an in-person group (14). Group cohesion and therapeutic alliance are core components for a successful group intervention (15), hence it is important to explore the nuance of how technology affords and constrains therapeutic interaction to improve intervention performance.

### The Present Study

This study explored the role of technology affordance in therapeutic interactions in ICT-delivered group psychotherapy with and without a physical exercise component. Particularly, it examined how ICTs afford and constrain group cohesion and therapeutic interactions. It would deepen our understanding of technology affordance in the relational process in digital group among other tasks related to intervention performance, such as logistic management and technical support. This study adopted a multiple triangulation approach to data sources, investigators, and methods, to provide a holistic picture and enhance the findings’ credibility and validity (16).

## Research Design and Methods

We adopted multiple sites, multiple investigators, and multiple methods design. Qualitative methods employed in the study included on-site observation, focus groups, and clinical notes. All voice recordings and qualitative data were stored in computers accessible only to the research team. This study was approved by the Human Research Ethics Committee of the University of Hong Kong (references: EA2003001A and EA200132).

### Context

In response to the suspension of face-to-face social services for older people during the COVID-19 pandemic (17), partnered community centers in a community-based holistic mental health care program for older people at risk of or with mild-to-moderate depressive symptoms (JC JoyAge) (18) delivered online groups to their service users, such as acceptance and commitment therapy (ACT) combined with exercise and cognitive behavioral therapy for insomnia (CBT-I). All sessions were conducted through Zoom, and a dedicated WhatsApp group was created to facilitate communication outside group time and remind participants to attend the session.

### On-Site Observation

F.H.C.W. conducted on-site observations at 2 ACTs with exercise groups, attended the Zoom sessions and took research notes while observing the interactions in the session. F.H.C.W. was introduced to the participants but did not interact with them.

### Focus Group

#### Participants

Purposive sampling was applied to identify focus group participants. We asked social workers to invite older people who participated in online groups to join our focus groups to share their experiences. We included older people who joined but could not complete the group entirely online to account for the diverse experience in the population. They were service users of a community-based holistic mental health care program for older people at risk of or with mild-to-moderate depressive symptoms (JC JoyAge) (18). Inclusion criteria for the JC JoyAge project were community-dwelling older people aged 60 years or older with moderate to moderately severe depressive symptoms, as indicated by scoring 5 or above in the Patient Health Questionnaire-9. Participants in the ACT with exercise group also exhibited chronic pain for more than 3 months, and those in the CBT-I group displayed sleep problems.

#### Procedure

With participants’ consent, we obtained their demographic information from social workers, including age, gender (male or female), marital status, education level, living arrangements, housing conditions, and physical impairment. An interview guide was developed to ascertain participants’ experiences of attending these ICT-enhanced intervention groups (see Supplementary Material). D.K.Y.L. and F.H.C.W. conducted the in-person focus groups between April and August 2020. The focus groups were conducted in community centers and lasted approximately 1 hour. All focus groups were conducted in Cantonese, audiotaped, and transcribed verbatim without personal identification. Participants provided informed consent to take part in the focus groups and to be recorded. We conducted ongoing focus group until data saturation. A preliminary codebook was developed based on the first 2 focus groups to guide data analysis for the subsequent focus groups. We decided to cease data collection after the fourth focus group, where no new information was revealed.

### Clinical Notes

Two clinical psychologists and one physiologist who provided the ACT with exercise, and one clinical psychologist who provided the CBT-I were invited to share their anonymized clinical notes.

### Data Analysis

Thematic analysis was used to analyze the data (19). D.K.Y.L., F.H.C.W., E.L.Y.W., and L.S. familiarized themselves with the data by reading the observation notes, transcripts, and clinical notes. The authors jotted down initial ideas and systematically coded the observation notes and 2 focus group transcripts to identify content relevant to the research question. The authors compared, discussed, and modified the codes before moving on to the remaining transcripts and clinical notes. During the process, new codes were generated, and some existing codes were modified. D.K.Y.L. and F.H.C.W. organized the codes into broader themes, which were then brought to a discussion with E.L.Y.W., L.S., and M.C. The authors reviewed the themes, making connections with the associated extracts and the overall data set. For example, the codes “camera field issues,” “screen size,” and “overlapping voices” were initially grouped under a theme named “capture-broadcast dilemma” because they were all related to how Zoom captured and broadcasted a scenario. During this phase, the authors reorganized the codes into the themes “seeing–be-seen dilemma” and “speaking–hearing dilemma” to distinguish how technology afforded and constrained visual and audio communication. D.K.Y.L. and F.H.C.W. further refined names and definitions for each theme. All authors agreed on the final themes. Quotes relevant to the identified themes were translated to English and are reported in the Results section. The translation was conducted by D.K.Y.L. and cross-checked by E.L.Y.W. The Standards for Reporting Qualitative Research checklist (20) is presented in Supplementary Material.

### Reflexivity

D.K.Y.L., E.L.Y.W., L.S., and M.C. have psychology training backgrounds, and F.H.C.W. is trained in journalism and communications. L.S. and M.S. also have experience delivering teleconference training programs and interventions to older people. We noted our a priori assumptions about ICT-delivered interventions to aid reflexibility in analysis (21). We assumed that interactions in ICT-delivered interventions could be different from face-to-face interventions, and these were affected by technology affordances, that is, the functions on Zoom and WhatsApp. Steps taken to reduce the impact of personal biases included having 2 researchers in each focus group and ensuring that each transcript and observation note was coded independently by 2 researchers.

## Results

Four focus groups were conducted with 22 older people who participated in 2 ACT with exercise groups (*n* = 11) and 2 CBT-I groups (*n* = 11) conducted between March and August 2020. Table 1 presents participants’ demographic characteristics. Their mean age was 72.6 years (standard deviation = 7.2), and most were female. Around half were married, living with another person, and had a primary school education or below. Around two thirds lived in private housing. They had high mobility, good hearing, and vision. Participants were existing members of their corresponding community centers and had previously participated in center-based face-to-face group activities. Five participants from the ACT with exercise group only joined 2 sessions on physical activity online because they had difficulty managing the technology.

**Table 1.** Participants’ Demographic Characteristics (*N* = 22)

Variable	Mean	*SD*	*N*	%
Age	72.59	7.20		
Gender (female)			19	86.4
Marital status				
Married			12	54.5
Single/divorced/widowed			7	31.7
Missing			3	13.6
Education level				
Primary school or below			13	59.1
Secondary school			4	18.2
High school			2	9.1
University or above			3	13.6
Living arrangements				
Living alone			6	27.3
Living with another person			12	54.5
Missing			4	18.2
Housing conditions				
Private housing			15	68.2
Public housing			3	13.6
Missing			4	18.2
Physical impairment				
Mobility (0–2)	0.53	0.51		
Hearing (0–2)	0.21	0.42		
Vision (0–2)	0.11	0.32		
Intervention received				
Acceptance and commitment therapy with exercise			11	50.0
Cognitive behavioral therapy for insomnia			11	50.0

We identified 3 challenges: (1) the seeing–be seen dilemma, (2) the speaking–hearing dilemma, and (3) blurred therapy–home boundary, and 2 solutions: (1) maneuvering layouts and collaborative tools, and (2) cross-platform mediated strategies (Table 2).

**Table 2.** Themes and Codes

Themes	Codes
Challenges	
Seeing–be seen dilemma	Camera field issues
	Screen size
Speaking–hearing dilemma	Overlapping voices
	Interactions with group members
	Interactions with interventionists
Blurred therapy–home boundary	Privacy issues
	Difficulty concentrating
	Convenience
Solutions	
Maneuvering layout and collaborative tools	Zoom layouts
	Collaborative tools
Cross-platform mediated strategies	Supplementary materials
	In-person training or support

### Seeing–Be Seen Dilemma

The seeing–be seen dilemma occurred in the exercise groups and depicted participants’ struggle between observing the interventionist’s on-screen instructions and presenting their full-body movements to the interventionist for guidance in front of the camera. The narrower field of view and uncontrollable camera from the interventionist created a different communication experience for participants. Unlike in-person interactions, the camera’s field of view was narrower and fixed, excluding anything outside the camera’s field of view from participants.

When the interventionist demonstrated full-body exercises and parts of her body were not in view of the camera, some participants tilted their heads trying to peek at what was outside the camera’s field of view as if they were in an in-person session. Some participants constantly adjusted their position, switching between sitting closer and further away from the device. One participant reflected that the display on his phone was too small to see the interventionist when he moved away from the camera to show the interventionist his movements. (Observation notes)

When participants needed to use the same technology to present themselves to others with such a novel experience in mind, they tended to move further away from the camera so that the camera could capture their whole body. However, some struggled to capture their entire body because of environmental limitations, such as being in a small room. Some who sat further from the camera found it challenging to observe what the interventionist was doing because her image on the screen appeared very tiny.

To be honest, I think she [the interventionist] could only see my upper body, but not the movement in my lower body. The device can only capture certain parts of the [visual] field—like if I put it here, it captures my upper body, so I moved further and further away from the camera, but she still couldn’t see my lower body. (Participant A, group 1)

The camera’s limited field of view made it difficult for the interventionist to determine whether the participants were doing the exercise correctly because some body parts were out of sight. The interventionist used more oral instructions to ensure participants carried out the exercise as correctly as possible.

The angle of the camera depends on the participant’s surroundings, and they may not be able to show their whole body or the exercise (eg, squatting but won’t be able to see the legs). This can be resolved by explicitly telling the participants which muscles they’re supposed to be feeling or working on and asking them to let me know if they felt more pain or have any questions. Since we’re also sitting further back to demonstrate the exercise better, we must look tiny on their screen. To ensure we’re all doing the same exercise at the same time, we need to describe the exercise and clearly explain each step. (Interventionist C)

### Speaking–Hearing Dilemma

In both psychological and exercise groups, simultaneously hearing others clearly and being heard also posed a dilemma. While participants are usually encouraged to share in a therapeutic group, overlapping voices could disrupt participants’ ability to hear the interventionists or other group members. The technology affords a key solution to avoiding overlapping sounds through the “Mute all” function and the interventionist’s ability to moderate the interaction.

When two or more people were speaking simultaneously, Zoom spontaneously suppressed all but one voice. This interrupted the flow of conversation. Participants were then all muted and the interventionist invited them to speak in turn. The interactions were usually limited to between the interventionist and a certain participant at a time. Fewer interactions among the participants were observed. (Observation notes)

Although participants agreed that being put on mute allowed them to listen to the interventionist or a particular group member clearly, they found it difficult to get to know other participants because of insufficient opportunities or time to talk to each other. Also, the interventionist might not be able to notice and address their concerns immediately. Some participants preferred face-to-face sessions because they allowed more flexibility in communicating with each other.

I think [the interventionists] can mute us all when they are talking. Then we can listen to them clearly without disruption from other people’s voices. If we have something to say, we can raise our hands. This will be the best. (Participant A, group 1)

Sometimes when we couldn’t see or hear [the interventionist], we raised our hand for help… but sometimes she couldn’t see me since there were many of us… I could only keep raising my hand until she noticed me. It’s not easy for her to pay attention to so many of us. (Participant A, group 3)

It will be better face-to-face. At least we can interact with each other, and if you don’t know how to do something, the person sitting next to you can tell you... This can’t happen online... The interventionist spoke to us in turn, she invited someone to answer or speak. (Participant C, group 4)

Despite greater control over interactions with participants, the “mute all” function was believed to have affected the sense of engagement in the group because of the lack of interaction between participants.

Because participants could not provide immediate feedback, the interventionists needed to check their status constantly to provide any information about adjusting the intervention.

Although the whole group cheered and clapped for a particular member, he or she could only hear the sounds from me since everyone else was muted. This has somewhat affected the sense of group cohesion. (Interventionist A)

Apart from proactively checking with the participants, the regular breaks scheduled throughout the session allowed them to give feedback and raise questions about the movements. It was helpful to know each participant’s sensation as they were performing the exercise so that I could provide specific instructions or modify the movements to ensure that they would not injure themselves. (Interventionist C)

### Blurred Therapy-Home Boundary

An ICT-delivered intervention joined 2 venues with different natures—the therapy room and participants’ homes. A therapy group can be considered a “private sphere” in which participation is restricted to permitted persons. However, study participants tended to join the intervention group in living spaces shared with other family members because they lacked sufficient privacy, a very common phenomenon in Hong Kong. Therefore, the home setting introduced a “public sphere” in the therapy. The camera transmitted the picture and sound of the public sphere into the private sphere and, at the same time, conversely, broadcast the sight and sound from the private sphere to the public sphere. The activities undertaken in the 2 spheres are usually different, but the online session encouraged the blurring of this boundary. The challenge then occurs when participants undertake activities at home that are normally considered out of place, for example, when interruptions from the background environment disrupted audio-visual communication.

When participants unmuted themselves to speak, there were noises from the background, such as television and conversations among other family members. It was difficult for other people to hear what the participant said. (Observation notes)

Intersections of family living spaces affected participants’ ability to concentrate on the interventions. Against the background of studying and working from home during the COVID-19 pandemic, family members were more likely to stay at home all day. The presence of family members may also jeopardize participants’ sense of security, particularly when the discussion was related to family issues.

There were three of us at home. I was in my room and they were in the living room. We sat separately from each other. Since my grandchildren attended online classes from home, all three of us were in teleconferencing and it can be noisy sometimes. (Participant D, group 3)

When someone at home asks you for help to get something, you will walk away to do so and miss out on what the interventionist was saying. You’re less engaged. Sometimes you just walk away to grab a cup of coffee during the session. (Participant F, group 2)

I would whisper; I don’t want them [family members] to hear me. My son was at home, and I kept my voice down so that he wouldn’t hear me. (Participant C, group 1)

Nonetheless, one participant expressed that the familiarity of her surroundings allowed her to speak her mind more easily and remain engaged in the group, given that no other people were around. Another participant was a carer for his wife with dementia and appreciated the chance to join the intervention online, enabling him to care for his wife and his own mental well-being simultaneously.

It’s probably because when you’re at home, and sometimes family members are not around, you feel more relaxed and can open up. On the contrary, the face-to-face environment [in the community center] is unfamiliar to you. Staying at home seems to be better since you will be less affected [by the relative unfamiliarity of the surroundings]. (Participant F, group 1)

Interventionists noticed that some participants did not or were unable to use earphones, which raised concerns about being overheard by participants’ family members and reduced their sense of security. In a group setting, this also risks exposing other participants’ privacy.

Family members could overhear the discussion in the group and sometimes interrupted participants’ sharing. They attempted to clarify or explain the issues the participants raised. This affected participants’ willingness and comfort to share their thoughts. Participants were encouraged to join the group from somewhere where they could speak freely. We may arrange for them to join the group at the community center. (Interventionist B)

### Maneuvering Layout and Collaborative Tools

The interventionists changed the layout in Zoom according to the intervention activities. The share screen, spotlight video, and gallery view are some features in Zoom that interventionists commonly employed in their sessions. During screen sharing and video spotlighting, the shared content and the spotlighted video are on the larger display for all participants, respectively. On the other hand, the gallery view feature allows participants to see thumbnail displays of all participants in a grid pattern.

The interventionists used the share screen function to show the PowerPoint slides and worksheets to the participants. They also spotlighted themselves when demonstrating in-class or homework exercises, such as progressive muscle relaxation and physical activity, to make sure participants could see them in a larger display. They stopped sharing the screen or removed the spotlight after the demonstration and reminded participants to switch to gallery view if they had not done so so that they could all see each other. (Observation notes)

Participants appreciated being able to see each others’ faces without masks during the pandemic, which helped them form connectedness with each other. They also found the display name on Zoom helpful in getting to know each other.

Every time the interventionist asked a question, she invited one of us to answer first and then asked that person to invite the next person to answer by his or her name. You had to look at their [display] names to choose the next person, and gradually you could remember their names. (Participant B, group 1)

The interventionists also reduced activities that required physical interactions, such as chair sculpture in ACT, which requires participants to put their chairs on top of other participants’ chairs. They introduced activities through Zoom that encouraged instantaneous interactions. For example, by enabling the annotation functions, such as the screen sharing whiteboard, participants could draw on the screen together simultaneously, which all participants could see.

We tried different activities with the screen sharing to keep participants engaged, such as matching the questions and answers shown in two columns, or showing a photo in PowerPoint and asking participants to circle the wrong food items to eat before sleeping, etc. (Interventionist D)

### Cross-Platform Mediated Strategies

Interventionists also adopted cross-platform mediated strategies to resolve the challenges by communicating and delivering supplementary materials through different media, including video streaming and printed materials.

The interventionists sent video links and audio files to the WhatsApp group during the session. They demonstrated how to access the videos and recordings on Zoom by holding the mobile phone closer to the camera. They encouraged participants to follow the instructions in the videos and recordings at home. (Observation notes)

Participants appreciated these supplementary materials so they could revisit and familiarize themselves with the exercise movements outside class. Therefore, they could sit further away from the camera and focus on demonstrating their movements to the interventionist for feedback. They also suggested using printed supplementary materials so they were more familiar with printed forms of materials, and print copies would not be affected by access to the internet.

We were given a link [that shows a video of the exercises taught in class], so we can watch it over and over again to make sure we did the movement correctly. (Participant B, group 1)

The interventionist demonstrated how the relaxation exercise is done in the video, guiding us on how to breathe and relax. (Participant A, group 4)

Printed instructions would help… like a book that includes pictures of a person demonstrating the movements with labels of “left leg” and “right leg” on the side pointing to the person. (Participant C, group 2)

Participants also found printed guidelines and in-person training on teleconferencing technology provided by social workers ahead of the online groups helpful in navigating the devices and software.

[The printed guidelines] allow us to follow paper instructions for reading certain pages at home...The social worker was great and photocopied [the printed guidelines], which included first steps like the pages to look at and which [application] button to press. There was a small book with a few pages photocopied for us, and she would tell us to turn to a specific page or press here and there [on the application] to turn on the device.

The interventionists acknowledged the need to provide technical training before the ICT-delivered interventions and considered it an opportunity to build rapport with participants individually. They also noticed that some participants were empowered through learning how to use ICT so they could become skilled at technology or acquire a novel skill.

For Zoom groups where participants don’t know the therapist before the group, it’s a good idea to arrange a ‘pre-group phone call or Zoom call’ individually to get to know each participant better first. It’s also an opportunity for them to try and sort out any technical difficulties in advance. (Interventionist A)

In the first Zoom session, some participants were visibly anxious and explicitly expressed their worries about managing the hardware (eg, tablet) or software (eg, Zoom). However, with more practice, some participants were able to master the new skill which may have helped them de-stigmatize their prior beliefs that “I can’t learn anything new when I’m old”. (Interventionist D)

## Discussion and Implications

This study explored the challenges of using ICT in delivering group psychotherapy with and without exercise components using a multiple triangulation research approach and solutions to them. From a technology affordance perspective, we identified 3 challenges and 2 potential solutions that were synthesized across different modalities in complex intervention settings and for older users. Five technology affordances—interactivity, portability, temporality, persistence, and multimediality appear to be relevant to therapeutic interactions in digital intervention, largely echoing the findings from Yeshua et al. (10). Table 3 summarizes their relations to the identified challenges and solutions. Central to therapeutic interaction is interactivity, which is defined as “interaction-generating functions of the digital app’s interface, including the ability to comment, meta-voice, provide feedback, and engage” (10). It acts with other technology affordances to facilitate or hinder interactions.

**Table 3.** Relationships Between Technology Affordance, Challenges, and Solutions

	Interactivity	Portability	Temporality	Persistence	Multimediality
Challenges					
Screen-camera dilemma	x	x			
Speaking–hearing dilemma	x				
Blurred therapy–home boundary	x	x			
Solutions					
Maneuvering layout and collaborative tools	x				
Cross-platform mediated strategies	x	x	x	x	x

While the screen-camera dilemma, the dilemma in hearing, and the blurred therapy–home boundary arose from innate characteristics of relaying interventions on a virtual platform, the particular intervention content of the psychotherapy and exercise program meant that they have a unique relational affordance. Portability is a related technology affordance, referring to the ability to transport device and use it regardless of the user’s location (22). The screen-­camera dilemma was more salient for participants in the exercise program. Exercise interventions required participants to observe and present full-body movements, and the camera’s limited field of view and small on-screen display pose more significant challenges to participants. Participants’ living space may also affect how much the camera could capture. The interventionist was also challenged to evaluate participants’ responsiveness to the exercise when the camera could not capture parts of their bodies. Similar constraints have been reported in individual teleconferencing psychotherapy, when the therapist has limited access to nonverbal behaviors or facial expressions when the client sits too close or too far from the camera, respectively (23). Limited verbal and nonverbal cues challenge therapists to evaluate the client’s responsiveness to the treatment plan, quality of therapeutic alliance, and the resonance of hypotheses being investigated (24). Therefore, as our findings demonstrate, the interventionists constantly needed to check their progress and feelings with participants.

On the other hand, the blurred therapy–home boundary appeared to be more disruptive in psychological interventions. Restricted living space in Hong Kong meant that participants’ therapy-home setting was more likely to intersect with family members’ private space. The median floor area of accommodation of domestic households was 40 m^2^ and the median per capita floor area was 16 m^2^ (25). Participants tried to prevent family members from interrupting and being interrupted by teleconferences. Occasionally, they also stepped out of sessions to fulfill their care tasks. Compared to the exercise program that mainly focuses on body movement, psychotherapy involves discussion of personal concerns and the blurred boundary raises privacy issues, jeopardizing participants’ sense of security and willingness to share these personal or sensitive issues. In individual teletherapy, some clients report resorting to receiving therapy in their car or while out walking to avoid being overheard (23). If participants in a group intervention do not wear earphones, other participants’ privacy might also be challenged, thus potentially affecting therapeutic alliance and group cohesion. Nonetheless, some may find it comfortable to speak their minds in a familiar environment, such as the home setting where they spend most of their time. A protected space at home could potentially facilitate the intervention when arranged properly.

Synchronous and asynchronous technologies constitute dissimilar affordances, which should be utilized by therapists to facilitate interventions. Findings suggest that, while Zoom and WhatsApp both allow multiple users to communicate, their differences in functional features shape distinct power relationships and engagement mechanisms on the platforms. Videoconferencing apps’ functionality affords synchronous interactions, rapport building, and the introduction of information using different layouts. According to theories of social presence, live visual and audio cues in videoconferencing enhance feelings of connectedness and support in a group setting (14,23). Furthermore, enabling annotation functions such as the screen-sharing whiteboard allowed participants to engage in group activities; however, functions like “mute all” gave power to interventionists to dictate interaction dynamics in the videoconference. The absolute power of the interventionist may limit interactions among group members. Because the “mute” function is a binary function that allows or disallows a user to be heard by everyone, interventionists muting and unmuting participants to reduce background noise or invite them to speak may disrupt the usual flow of conversation between participants. Group cohesion was undermined by the centralization of communication, resonating with the findings of Lopez et al. (14).

Nonetheless, technologies for asynchronous communication may compensate by sustaining uninterrupted conversations with more equal power between interventionists and participants. Two related technology affordances are temporality and persistence. Temporality refers to how digital interaction over time is experienced by individual users (26), and persistence refers to the ability to access the original display of communication after the actor has completed his or her presentation (27). The high temporality of the instant messaging app enables ongoing communication outside the group session, allowing participants to ask the interventionists questions and chat with one another (10). Because messaging apps functionally keep the conversation record on the platform in normal circumstances, participants can trace what have been said. This feature can be particularly useful for participants who were less engaged in the synchronous sessions. Messaging app also has high multimediality that offer a wide range of multimedia features, such as voice and video calls, and sending pictures, emojis, and audio and video messages (22). It enables therapists to bypass the screen-camera dilemma by sending course-related notes and homework in links, images, audio recordings, and videos that participants can revisit anytime. From the participants’ perspective, technology affordance means more than the potential of the technology in hand, but also their perception and familiarity with the tools. Therefore, coupling ICT-delivered interventions with communication methods that participants are more familiar with, such as printed materials, telephone calls, and face-to-face education sessions, is useful in helping participants to build their confidence in technologies. Therefore, consistent with Yeshua-Katz et al. (10), combining and matching the affordances of different ICTs to the nature of the intervention and participants’ characteristics could enhance intervention performance and resolve the digital divide.

### Clinical Implications

Online interventions have emerged as a solution to the challenges brought by the COVID-19 pandemic, encouraging therapists to develop knowledge that can fundamentally change the mode of future intervention delivery. ICT-delivered intervention can provide greater accessibility to social care for individuals who cannot attend in-person sessions. Bearing in mind the blurred therapy–home boundary, therapists could work with participants to optimize their physical environment to reduce distractions and preserve privacy for themselves and other participants. Interventionists could set platform-specific rules to avoid overlapping voices and encourage smoother interactions among participants, such as raising hands to speak and asking participants to invite the next person to speak. Before the interventions, interventionists could arrange individual ICT-based or in-person meetings with participants to build rapport and get to know their digital literacy, thus ensuring adequate support for operating ICT, such as printed materials or in-person training. Interventionists should familiarize themselves with the affordance of different ICTs so that they can modify intervention activities accordingly. For example, screen sharing and whiteboard functions in Zoom enabling participants to add annotations may be useful to engage multiple participants and enhance group cohesion. Interventionists could also consider employing multiple platforms to achieve intervention goals. In exercise interventions, for instance, sharing supplementary material such as videos of exercise via instant messaging apps or print material may be particularly helpful for participants to revisit the exercise without time restriction and the camera’s limited view of the field.

### Limitations

This study has several limitations. Participants included in this study might have higher digital literacy and motivation given their willingness to join in psychosocial interventions using a delivery mode different from usual practice. Recent census survey revealed that 68% of older people in Hong Kong own a smartphone and 49% of them know how to use a personal computer. They reported mainly using the Internet for communication, information searching, and online entertainment (28). While digital literacy was not measured, participants’ reflections on their experiences, particularly the challenges they faced, suggest their digital literacy and experience with ICT-delivered activities varied. In particular, the ICT-delivered mode of one ACT with exercise group was discontinued because participants could not manage the technology. Study participants were older people with depressive symptoms, whose experience may not be generalized to those without depressive symptoms, who may have better physical health and family relationships (29,30). However, findings from this purposive sampling would benefit the development of digital interventions for older people with mental health needs. Additionally, some findings appear applicable to other settings and populations. For example, the seeing–be seen dilemma due to small screen size could also occur in a younger population undergoing individual teleconferencing psychotherapy, where nonverbal communication may be out of sight when the therapist and client sit too close or too far away from the camera.

Moreover, the study focused on group therapy, Zoom, and WhatsApp. Although some challenges and strategies identified in the study may be applicable across intervention modalities and ICTs, technology affordance is relational and functional-related. Future studies can explore the unique technology affordances of other applications and intervention modalities, such as psychoeducation that involves more information provision but less personal sharing, to identify how applications can be best adapted to improve older people’s experience of different types of interventions.

## Conclusion

The challenges and possibilities synthesized across group psychotherapy and exercise programs in complex intervention settings for older users can be understood from technology affordance perspective: interactivity, portability, temporality, persistence, and multimediality. The dilemmas of seeing–be seen and blurred therapy–home boundary were more prominent in the exercise program and psychotherapy, respectively, whereas the speaking–hearing dilemma affected both intervention modalities. These challenges originated in the inherent qualities of conveying group interventions on a virtual platform, influencing therapeutic interactions and group cohesion. Interventionists maneuvered layouts and collaborative tools in teleconferencing media according to intervention goals and used cross-platform strategies to address these. Technology affordance can be used to guide intervention development. Matching the affordances of various ICTs with the characteristics of the intervention and participants can improve intervention efficacy and avoid an unnecessary digital divide.

## Supplementary Material

igad063_suppl_Supplementary_Material

## Data Availability

Data are available upon reasonable request.
