# Supplementary material for: Technology Affordance in an Information and Communication Technology Delivered Group Psychotherapy and Exercise Program for Older People With Depressive Symptoms: A Multiple Triangulation Qualitative Study
Source: Innov Aging. 2023 Jul 7;7(6):igad063. doi: 10.1093/geroni/igad063 (PMC10430789; doi:10.1093/geroni/igad063)
Supplement: igad063_suppl_Supplementary_Material [file igad063_suppl_supplementary_material.docx]

*Innovation in Aging* Online Supplementary Material: Dara Kiu Yi Leung, Frankie Ho Chun Wong, Edwin Lok Yan Wong, Lesley Sze, Melissa Chan, Tianyin Liu, Annabelle Pui Chi Fong, Wai Wai Kwok, Angie Kwan Yu Shum, Gloria Hoi Yan Wong, & Terry Yat Sang Lum. Technology affordance in an information and communication technology-delivered group psychotherapy and exercise program for older people with depressive symptoms: A multiple triangulation qualitative study.

**Interview Questions**

1. How did you feel about using ICT to participate in group activities?
   1. The process of using ICT
   2. The process of expressing yourself and receiving instructions
   3. The process of communication with other participants and tutors
2. What factors make it easier/difficult for you to participate in online group activities?
3. Under what circumstances can you enjoy group activities or feel yourself engaged in group activities? What is your reaction or performance when you are very involved in group activities?
4. When the other group members are not physically around, how do you maintain your participation in the team or solve the difficulties you encounter?
5. In terms of learning and participation, what is the difference between online group activities and in-person participation?
6. How do you think you can improve the entire online learning process and help you exercise outside of your group?
7. How does ICT affect your participation in the group?
8. How do you feel about sharing your experience in front of the camera?
9. How do you seek the assistance of a therapist in a video conference? Did the whole process go smoothly? Why?
10. How long can you concentrate on the video conference? What factors affect your concentration?
11. How do you solve the problems encountered when learning sports movements in a video conference?
12. How often do you exercise? What factors affect your exercise plan?
